# Mutation spectrum analysis of *DMD *gene in Indonesian Duchenne and Becker muscular dystrophy patients

**DOI:** 10.12688/f1000research.73476.2

**Published:** 2023-02-07

**Authors:** Ery Kus Dwianingsih, Kristy Iskandar, Sunartini Hapsara, Chun Ping Liu, Rusdy Ghazali Malueka, Gunadi, Masafumi Matsuo, Poh San Lai

**Affiliations:** 1 Genetics Working Group, Faculty of Medicine, Public Health and Nursing, Universitas Gadjah Mada, Yogyakarta, 55281, Indonesia; 2 Dr. Sardjito General Hospital, Yogyakarta, 55281, Indonesia; 3 Department of Anatomical Pathology, Faculty of Medicine, Public Health and Nursing, Universitas Gadjah Mada, Yogyakarta, 55281, Indonesia; 4 Academic Hospital, Universitas Gadjah Mada, Yogyakarta, 55291, Indonesia; 5 Department of Child Health, Faculty of Medicine, Public Health and Nursing, Universitas Gadjah Mada, Yogyakarta, 55281, Indonesia; 6 Department of Pediatrics, Yong Loo Lin School of Medicine, National University of Singapore, Singapore, 119228, Singapore; 7 Department of Neurology, Faculty of Medicine, Public Health and Nursing, Universitas Gadjah Mada, Yogyakarta, 55281, Indonesia; 8 Pediatric Surgery Division, Department of Surgery, Faculty of Medicine, Public Health and Nursing, Universitas Gadjah Mada, Yogyakarta, 55281, Indonesia; 9 KNC Department of Nucleic Acid Drug Discovery, Faculty of Rehabilitation, Kobegakuin University, Kobe, 651-2180, Japan

**Keywords:** Duchenne muscular dystrophy, Becker muscular dystrophy, DMD gene, mutation analysis, Indonesia, MLPA

## Abstract

Background: Duchenne muscular dystrophy (DMD) and Becker muscular dystrophy (BMD) are allelic disorders caused by mutations in the *DMD* gene. The full mutation spectrum of the DMD gene in Indonesian patients is currently unknown. Mutation-specific therapies are currently being developed, such as exon skipping or stop codon read-through therapy. This study was conducted with the aim of identifying the mutation spectrum of the *DMD* gene in Indonesia to guide future development and application of feasible therapeutic strategies.

Methods: This study is a cross sectional study that enrolled 43 male patients with a clinical suspicion of DMD or BMD. Multiplex ligation-dependent probe amplification (MLPA) reaction was performed to screen for the common mutations in the *DMD* gene.

Results: Out of 43 subjects, deletions accounted for 69.77% (n=30) cases, while duplications were found in 11.63% (n=5) cases. One novel duplication spanning exons 2 to 62 was identified. Deletion mutations clustered around the distal (66.67%) and proximal (26.67%) hot spot regions of the *DMD* gene while duplication mutations were observed solely at the proximal region. Two false positive cases of single exon deletion detected through MLPA were attributed to sequence mutations affecting primer ligation sites, confirming the need to validate all single exon deletions when using this screening method. Analysis of available maternal DNA samples showed that the rate of de novo mutations (48.15%) appears higher than expected in this population. Out of 31 patients who were classified as DMD based on clinical and genotype characterizations, 60.47% (n=26) of cases were suitable for exon skipping therapy.

Conclusion: This is the first comprehensive study showing the feasibility of implementing the MLPA method for routine screening of DMD patients in Indonesia. This is also the first study showing the potential applicability of exon skipping therapy in the majority of DMD cases in the country.

## Introduction

Duchenne muscular dystrophy (DMD) and Becker muscular dystrophy (BMD) are X-linked recessive disorders arising due to mutations in the *DMD* gene. ^1^ The *DMD* gene is one of the largest genes in the human genome with a size of more than 2 Mb. This gene spans 79 exons and codes for a 14 kb mRNA that translates a cytoplasmic protein called dystrophin. Due to this large size, mutation detection poses a challenge for routine molecular diagnosis in a clinical setting in many developing countries. Dystrophin protein interacts with other glycoproteins in cell membranes forming the dystrophin-glycoprotein complex (DGC) which stabilizes the membranes of muscle fibers. ^2^ The absolute absence of dystrophin leads to a clinical manifestation of muscle weakness from early childhood in DMD. Subsequent progressive muscle weakness leads to death before the third decade of life due to respiratory or cardiac failure. The presence of partially functional protein results in BMD, a milder phenotype of the disease. ^3^

DMD is the most frequently inherited muscle disease and reported to affect one in every 3,500 male births. ^1^ Indonesia is one of the largest countries in the world, with a population of 260 million. ^4^ This suggests that there should be many more DMD cases in Indonesia than the small number of cases that have been reported. ^5^ The true incidence of the disease and the underlying genetic variants are unknown in this region. Lack of awareness of the clinical features associated with the disease and limited use of molecular testing may lead to many undetected cases, resulting in an iceberg phenomenon of under-diagnosis and under-reporting of DMD cases.

Genetic analysis to detect *DMD* gene mutation is now a gold standard to diagnose DMD/BMD. However, this is not being performed regularly in developing countries like Indonesia. Gene deletions and duplications are reported to be the most common mutations in the *DMD* gene, encompassing more than 60% of cases. ^6^ Multiplex ligation-dependent probe amplification (MLPA) reaction, a quantitative PCR-based technique, is currently used to routinely amplify all 79 exons of the *DMD* gene to detect deletions and/or duplications in patients. ^7^ Meanwhile, the identification of point and small mutations for non-deletion or non-duplication cases remains challenging for resource poor labs because of the large number of exons in the *DMD* gene that requires sequence-by-sequence screening. As such, there is currently a lack of knowledge on the true mutation profile in Indonesian DMD/BMD patients. Future applications of mutation-specific molecular therapies, such as exon skipping, CRISPR-Cas9 mediated correction or stop codon read-through therapy, are currently being investigated for patients carrying mutations such as deletions, duplications, and small mutations. ^8^ Precise mutations analysis is thus necessary to apply the appropriate strategies to patients who are eligible for such treatments. ^8^ Thus, the objective of this study was to identify the spectrum of common deletion and duplication mutations in the *DMD* gene for clinically diagnosed patients in the Indonesian population using the MLPA technique. The study was carried out with a view to informing future therapeutic applications and developing specific approaches for disease management.

## Methods

### Patients

Forty-three male patients from Dr. Sardjito Teaching Hospital and Universitas Gadjah Mada (UGM) Academic Hospital, Yogyakarta, Indonesia were enrolled in this study, from 2017 to 2018. Clinical manifestations of DMD/BMD were found in the patients and supported by the findings of high serum creatine kinase (CK) levels through medical record data. Clinical diagnosis was made by qualified and experienced pediatric neurologists who are also trained molecular geneticists. Up to now, patients have been followed for their clinical management. Genetic analysis was performed based on approval of parents through signed written informed consent. The study protocol was approved by the Medical and Health Research Ethics Committee of the Faculty of Medicine, Public Health and Nursing, Universitas Gadjah Mada (KE/FK/0890/EC/2018) and National University of Singapore Institutional Review Board (N-19-102E).

### Immunohistochemical analysis

Out of 43 subjects, 23 subjects additionally consented to undergo a muscle biopsy procedure. Muscle biopsy in the form of formalin-fixed paraffin-embedded (FFPE) samples were sliced into 3 μm thickness, incubated, deparaffinized, and rehydrated. Antigen retrieval was performed using a Decloaking Chamber NxGen (BioCare Medical, USA) and immunostained with mouse monoclonal Dys-2 antibody (Novocastra, Leica Biosystem, Newcastle, UK, product code NCL-Dys-2, lot number 6062727) to detect dystrophin expression against carboxyterminal domain with dilution 1:25 in phosphate buffer saline (PBS). Diamino-benzidine (DAB) for visualization of positive cells was applied with a semi-automatic intelliPATH FLX (BioCare Medical, USA) according to the manufacturer’s instructions. Dys-2 expression was observed under a light microscope by an independent and experienced pathologist by analysing the expression of dystrophin in muscle membrane. Negative expression of Dys-2 is considered as indicative of DMD while faint or focal staining suggests BMD, with normal muscle tissue used as a positive control.

### Genomic DNA extraction

Genomic DNA was isolated from 3 mL of EDTA peripheral whole blood samples using Geneaid™ DNA Isolation Kit (Geneaid Biotech Ltd) according to manufacturer’s protocol.

### Genetic testing

MLPA analysis was conducted to screen all exons of the *DMD* gene using SALSA MLPA P034 and P035 probe sets (MRC Holland, Netherlands). ^7^ The procedure was performed according to the manufacturer’s protocol. Amplified products were separated using the ABI 3130xl Genetic analyzer and data were analyzed by Coffalyser software (MLPA analysis software by MRC-Holland). Patients with single exon deletion were confirmed using conventional PCR. DNA samples from normal healthy individuals were used as reference controls and included in every run. The primer sequences used to amplify the DNA were:

47F 5’-CGTTGTTGCATTTGTCTGTTTCAGTTAC-3’, 47R 5’-GTCTAACCTTTATCCACTGGAGATTTG-3’ (181bp); 51F 5’-GAAATTGGCTCTTTAGCTTGTGTTTC-3’, 51R 5’-GGAGAGTAAAGTGATTGGTGGAAAATC-3’ (388bp); 52F 5’-AATGCAGGATTTGGAACAGAGGCGTCC-3’, 52R 5’-TTCGATCCGTAATGATTGTTCTAGCCTC-3’ (113bp); and 65F 5’-ATTCTCAGAGGAAAAAGGACACTG-3’, 65R 5’-GTCTAACCTTTATCCACTGGAGATTTG-3’ (369bp).

PCRs were performed in a volume of 25 μL containing 2 μL of genomic DNA, 12.5 μL of 2× Go Taq green Master Mix, 1 μL of each primer, and 8.5 μL of nuclease-free water. The PCR cycling conditions of exon 47 were as follows: initial denaturation at 95 °C for two minutes followed by 35 cycles of denaturation at 95 °C for one minute, annealing at 50 °C for one minute, and extension at 72 °C for one minute. Amplification of other exons used the same PCR condition, however, the annealing time of exons 51 and 52, was 50 °C, meanwhile exon 65 was 55 °C. The amplified exons were then sequenced and analyzed for their mutation’s status. ^9^

Reading frame analysis was performed using online software LOVD exonic deletions/duplications reading-frame checker based on predicted translation of the DMD mRNA arising from the identified deletion/insertion (duplication) of the affected exons. Identified mutations were also compared against previously reported mutations using the UMD-DMD France mutation database ( http://www.umd.be/) and the Leiden Muscular Dystrophy pages mutation database accessed on June 6 ^th^, 2021.

## Results

### Demographic and clinical profile

All 43 subjects recruited for this study were male and showed clinical features of DMD/BMD such as difficulty in walking, muscle weakness, positive Gower sign at age of onset and increased CK levels in their blood samples. CK levels were elevated, ranging from 1,734 to 40,429 IU/L (mean 9,121.7 IU/L, two patients with unavailable data). The age of onset of the disease ranged from one to nine years old (mean 5.1 years old). Nineteen patients (44.18%) were wheelchair bound, with loss of ambulation starting between nine to 13 years old, with mean age at 11 years old ( Table 1).

**Table 1.** Demographic, clinical and genetic characteristic of Duchenne muscular dystrophy (DMD)/Becker muscular dystrophy (BMD) patients.

No	Patient ID	CK level	Age of onset	Age when wheelchair bound	Current age	Clinical features	IHC (dys2)	MLPA	PCR & sequencing validation for single exon deletion	Reading frame prediction	Geno-typing	Pheno-typing
1	dmd1	4906	6	10	11	waddling gait, tiptoe walk, prone to falls, unable to climb stairs, unable to sit without assistance, Gower sign (+), gross and fine motor skill delay, pseudohypertrophy	n/a	del 46–48		Out-frame	DMD	DMD
2	dmd4	4135	5	12	21	waddling gait, tiptoe walk, prone to falls, unable to climb stairs, unable to sit without assistance, Gower sign (+), gross motor skill delay, low birth weight, pseudohypertrophy, scoliosis	negative staining	no del/no dup		n/a	n/a	DMD
3	dmd5	10499	5	10	12	waddling gait, tiptoe walk, prone to falls, Gower sign (+), gross motor skill delay, cardiomegaly, scoliosis, ankle deformity	negative staining	del 53–54		Out-frame	DMD	DMD
4	dmd6	17388	4	not yet	5	unable to climb stairs and sit without assistance, Gower sign (+), cardiomegaly, mild scoliosis	n/a	Del 46–50		Out-frame	DMD	DMD
5	dmd8	2020	4	10	13	difficulty in walking, prone to falls, Gower sign (+), severe obstructive defect of respiratory tract, scoliosis	negative staining	del 17–43		Out-frame	DMD	DMD
6	dmd9	3932	5	10	14	difficulty in walking, prone to falls, Gower sign (+), scoliosis, pneumonia, knee contracture, malnutrition	n/a	del 45–52		Out-frame	DMD	DMD
7	dmd10	11152	5	not yet	7	tiptoe walk, prone to falls, difficulty in standing, Gower sign (+), mild scoliosis	faint staining	del 51	del 51	Out-frame	DMD	DMD
8	dmd11	16550	4	not yet	8	prone to falls, difficulty in walking, Gower sign (+), mild scoliosis, right coxae deformity	faint staining	del 46–51		Out-frame	DMD	DMD
9	dmd14	10846	5	not yet	7	waddling gait, prone to falls, tip toe walk, Gower sign (+), gross and fine motor skill delay, speech delay	negative staining	no del/no dup		n/a	n/a	DMD
10	dmd15	3125	8	not yet	9	Prone to falls, difficulty in walking and climbing, Gower sign (+)	negative staining	del 48–50		Out-frame	DMD	DMD
11	dmd16	8871	4	9	9	difficulty in walking, Gower sign (+), pseudohypertrophy, scoliosis	negative staining	del 45–52		Out-frame	DMD	DMD
12	dmd 17	6239	5	not yet	6	Difficulty in walking, prone to falls, Gower sign (+)	negative staining	del 52	del 52	Out-frame	DMD	DMD
13	dmd18	3255	6	10	13	Lost ambulatory, difficulty in walking, Gower sign (+), gross and fine motor skill delay, speech delay, right lower leg deformity	n/a	del 65	c.9540-9551 del CTGGCTGCTGAA	In-frame	BMD	DMD
14	dmd19	6924	5	not yet	6	difficulty in walking, Gower sign (+), pseudohypertrophy, muscle weakness	negative staining	dup 2–62 *		Out-frame	DMD	DMD
15	dmd20	12948	7	not yet	10	Difficulty in walking, prone to falls, waddling gait, Gower sign (+), gross and fine motor skill delay	negative staining	del 7–43		In-frame	BMD	BMD
16	dmd21	28060	6	not yet	7	Difficulty in walking and climbing, prone to falls, waddling gait, Gower sign (+), gross and fine motor skill delay, speech delay, muscle weakness, pneumonia	negative staining	dup 2–62 *		Out-frame	DMD	DMD
17	dmd22	no data	5	7	14	Difficulty in walking, Gower sign (+)	n/a	no del/no dup				DMD
18	dmd24	11433	5	Still ambulant	9	Difficulty in walking, Gower sign (+)	n/a	del 47–50		Out-frame	DMD	DMD
19	dmd25	NA	1	Still ambulant	6	Difficulty in walking and climbing, prone to falls, Gower sign (+), gross motor skill delay	n/a	del 49–52		Out-frame	DMD	DMD
20	dmd26	40429	6	Still ambulant	6	Difficulty in walking, Gower sign (+)	n/a	no del/no dup		n/a	n/a	DMD
21	dmd27	8961	6	Still ambulant	6	Difficulty in walking and climbing, tiptoe walking, prone to falls, Gower sign (+), muscle weakness	negative staining	no del/no dup		n/a	n/a	DMD
22	dmd28	4137	9	Still ambulant	9	Difficulty in walking, tiptoe walking, prone to falls, Gower sign (+), muscle weakness, scoliosis	negative staining	del 47	c.6808 del T (stop codon)	Out-frame	DMD	BMD
23	dmd29	8340	5	9	18	Difficulty in walking, tiptoe walking, prone to falls, Gower sign (+), difficulty in breathing, muscle atrophy, upper and lower extremity joint contracture, scoliosis, bedridden	n/a	no del/no dup		n/a	n/a	DMD
24	dmd30	8751	4	8	8	Difficulty in walking, prone to falls, Gower sign (+), gross and fine motor skill delay, inferior flaccid paresis	negative staining	del 51–54		Out-frame	DMD	DMD
25	dmd31	9603	5	10	10	Difficulty in walking, tiptoe walking, Gower sign (+), gross motor skill delay, speech delay, scoliosis	n/a	del 3–44		In-frame	BMD	DMD
26	dmd33	7482	6	Still ambulant	9	Waddling gait, prone to falls, difficulty in walking, Gower sign (+), gross motor skill delay, pseudohypertrophy	n/a	no del/no dup		n/a	n/a	DMD
27	dmd34	6746	2	9	10	Walking difficulty, tiptoe walking, Gower sign (+), muscle weakness, pseudohypertrophy, scoliosis	negative staining	del 49–50		Out-frame	DMD	DMD
28	dmd35	15448	6	Still ambulant	9	Walking difficulty, tiptoe walking, Gower sign (+), muscle weakness	faint staining	dup 14–17		Out-frame	DMD	DMD
29	dmd36	6857	4	Still ambulant	8	Walking difficulty, waddling gait, tip toe walking, prone to falls, Gower sign (+), pseudohypertrophy, lordosis	negative staining	del 18–47		Out-frame	DMD	DMD
30	dmd37	3337	5	Still ambulant	7	Walking difficulty, waddling gait, tip toe walking, gross motor skill delay, speech delay, Gower sign (+), pseudohypertrophy, muscle weakness, malnutrition	negative staining	del 56–74		Out-frame	DMD	DMD
31	dmd38	6607	7	7	8	Walking difficulty, tiptoe walking, Gower sign (+), muscle weakness, pseudohypertrophy, lordosis	negative staining	del 45–49		In-frame	BMD	BMD
32	dmd39	2678	5	10	11	Unable to stand up and walk, muscle weakness	faint staining	del 18–34		Out-frame	DMD	DMD
33	dmd42	1368	5	9	14	Prone to falls, difficulty in walking, ankle contracture, scoliosis	n/a	dup 2–44		Out-frame	DMD	DMD
34	dmd44	12324	5	Still ambulant	9	Difficulty in walking, Gower sign (+)	n/a	del 48–52		Out-frame	DMD	DMD
35	dmd47	13896	4	Still ambulant	6	Difficulty in walking, Gower sign (+)	n/a	del 49–50		Out-frame	DMD	DMD
36	dmd48	7800	2	13	14	Difficulty in walking, Gower sign (+)	n/a	del 3–7		Out-frame	DMD	DMD
37	dmd49	9569	9	Still ambulant	12	Difficulty in walking, Gower sign (+)	n/a	del 3–7		Out-frame	DMD	DMD
38	dmd50	7629	4	9	9	Difficulty in walking and climbing the stairs, easy to fall, Gower sign (+), muscle spasm	n/a	del 5–7		Out-frame	DMD	DMD
39	dmd51	7800	4	10	12	Tiptoe walk, prone to falls, unable to run, Gower sign (+), gross and fine motor skill delay, muscle spasm, Achilles’ contracture	n/a	del 3–17		Out-frame	DMD	DMD
40	dmd53	4865	5	Still ambulant	9	Tiptoe walk, prone to falls, difficulty in climbing stairs, Gower sign (+), muscle weakness, malnutrition	negative staining	del 51	del 51	Out-frame	DMD	DMD
41	dmd54	1734	6	9	11	Difficulty in walking, Gower sign (+), scoliosis	faint staining	dup 2–18		Out-frame	DMD	DMD
42	dmd60	13100	5	Still ambulant	10	Difficulty in walking, Gower sign (+)	n/a	del 49–52		Out-frame	DMD	DMD
43	dmd61	11367	5	Still ambulant	9	Difficulty in walking, prone to falls, Gower sign (+), gross motor skill delay, muscle weakness	n/a	no del/no dup		n/a	n/a	DMD

CK: serum creatine kinase; IHC: immunohistochemistry; MLPA: multiplex ligation-dependent probe amplification; n/a: not available; del: deletion; dup: duplication.

* Novel mutation.

Out of 43 patients, 35 (81.40%) cases of deletion and duplication could be detected using the MLPA method. Deletions accounted for 69.77% (30 cases), while duplications were found in 11.63% (five cases). Two patients who were initially found to have single exon deletion by MLPA turned out to carry small mutations after further investigation. In the remaining eight patients (18.60%), no deletion nor duplication was detected ( Table 1). The identified mutations were screened against two well-known DMD databases, namely UMD-DMD France and Leiden Muscular Dystrophy pages database. Two cases with a novel mutation were identified: dmd19 and dmd21 with duplication spanning exons 2 to 62 (c. (241)_(9433)dup) ( Table 1).

### Immunohistochemistry results

Out of 23 patients who underwent muscle biopsy, 18 cases (78.26%) showed negative expression of Dys-2 in muscle membrane, suggesting DMD, meanwhile the remaining five cases (21.74%) expressed faint and patchy staining, suggestive of BMD. However, eight discrepant cases were identified. Five faintly staining cases (dmd10, dmd11, dmd35, dmd39 and dmd54) had out-of-frame mutations, two cases (dmd20 and dmd38) with negative immunostaining were found to carry in-frame mutations, and one case (dmd28) with negative immunostaining carried an out-of-frame mutation but clinically had mild symptoms ( Table 2).

**Table 2.** Discrepancies among genotype, phenotype, and immunohistochemistry (IHC) staining.

Subject	Molecular genotype	Clinical phenotype	IHC results (protein)
dmd10	Out-frame (del 51)	DMD	±
dmd11	Out-frame (del 46–51)	DMD	±
dmd18	In-frame (del 65)	DMD *	n/a
dmd20	In-frame (del 7–43)	BMD	-
dmd28	Out-frame (del 47)	BMD *	-
dmd31	In-frame (del 3-44)	DMD *	n/a
dmd35	Out-frame (dup 14–17)	DMD	±
dmd38	In-frame (del 45–49)	BMD	-
dmd39	Out-frame (del 18–34)	DMD	±
dmd54	Out-frame (dup 2–18)	DMD	±

DMD: Duchene muscular dystrophy; BMD: Becker muscular dystrophy; n/a: not available; (-): negative staining; (±): patchy staining.

* Genotype-phenotype discrepancy.

### Deletion pattern in the *DMD* gene

**Figure 1.**
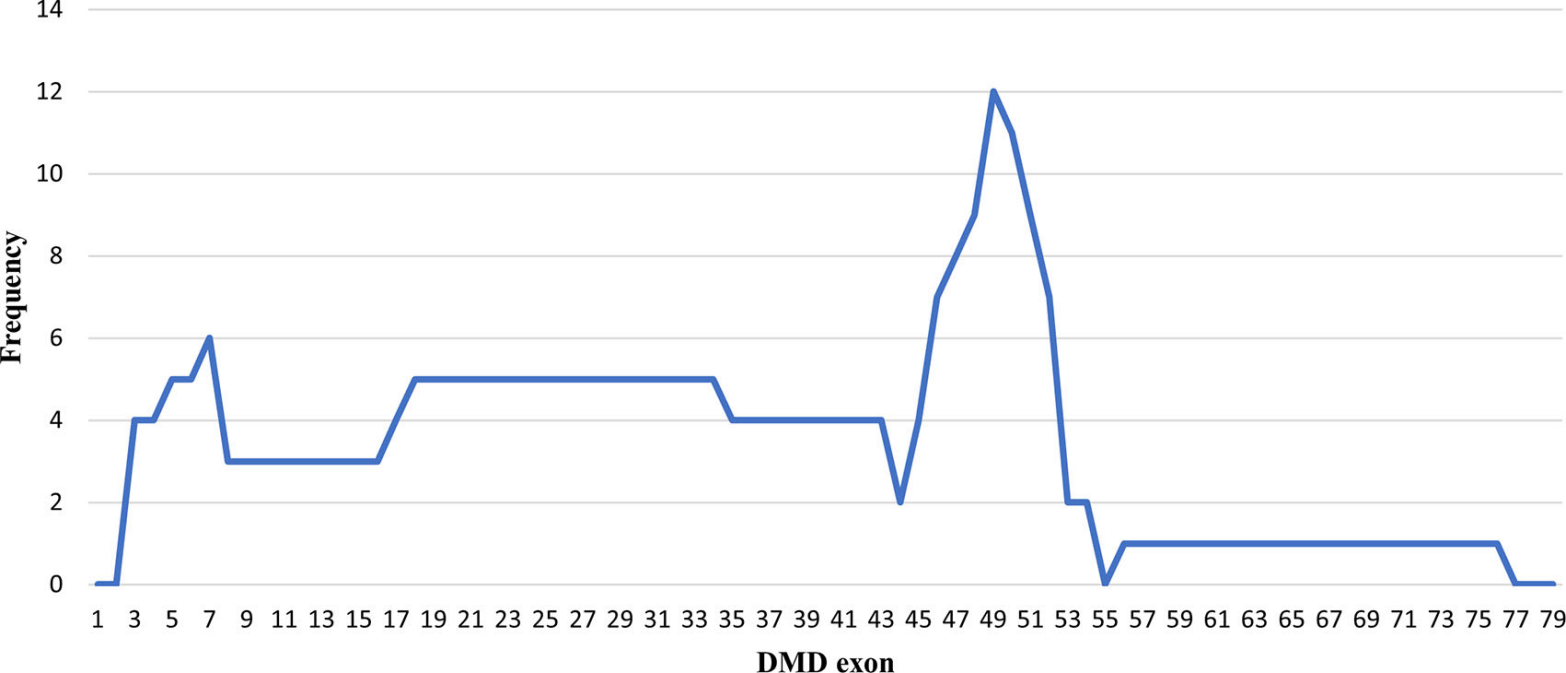
Frequencies of deletion of *DMD* gene exons in Indonesian Duchenne muscular dystrophy/Becker muscular dystrophy patients (n=28).

**Figure 2.**
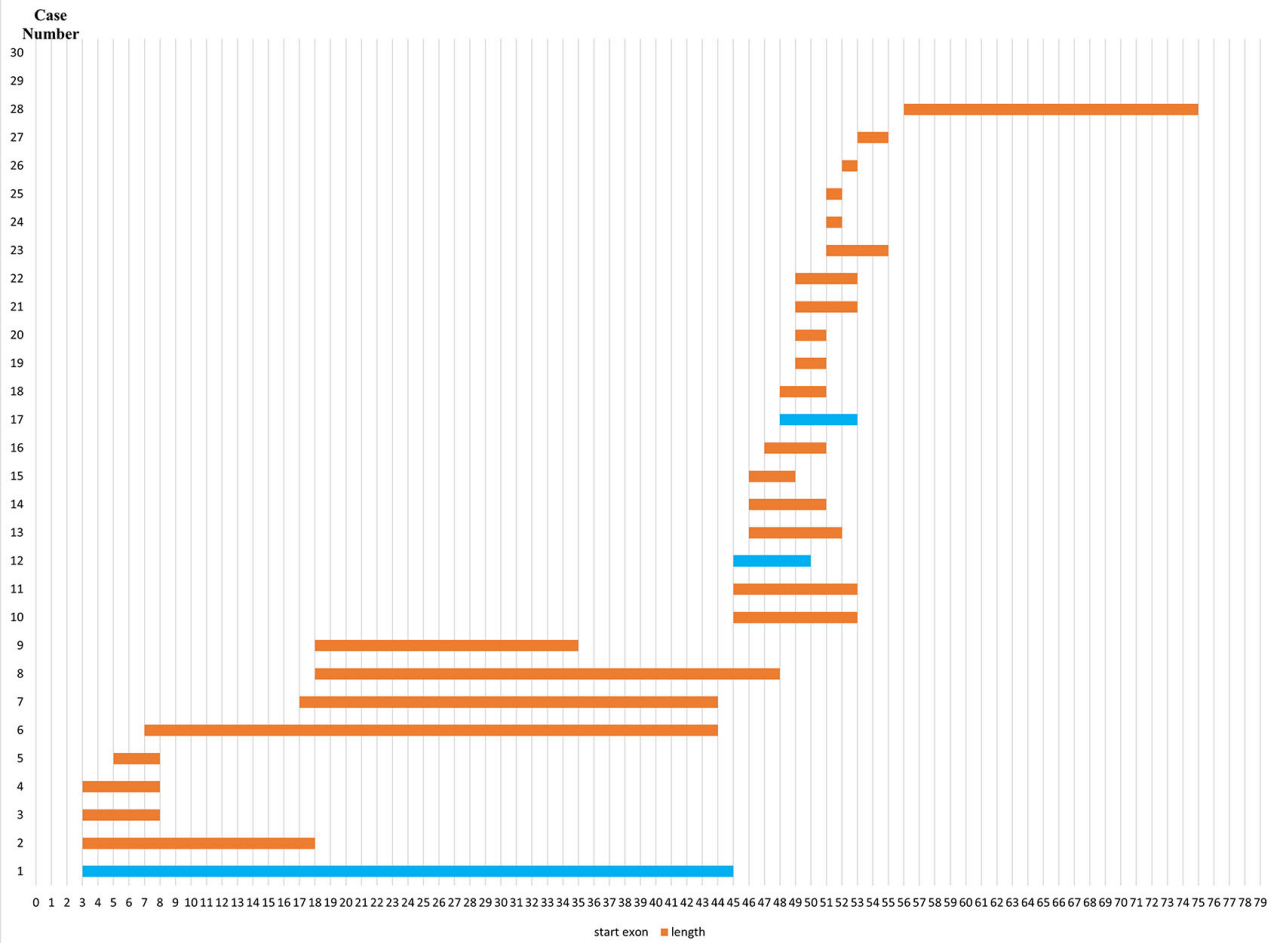
Distribution pattern of exon deletions in Indonesian Duchenne muscular dystrophy/Becker muscular dystrophy patients (n=28). Most exon deletions occurred in rod domain of *DMD* gene. Out-frame deletions indicated in orange boxes, while in-frame deletions indicated in blue boxes.

Initially, 30 deletions (69.77%) cases were discovered using MLPA, consisting of 25 cases with multi-exon deletions and five cases with single exon deletions of exon 47, exon 51, exon 52 and exon 65. Single exon deletion cases were further analysed by PCR. Three cases were confirmed to have single exon deletion of exons 51 or 52. After sequencing, a case with deletion in exon 47 turned out to carry a nonsense mutation (c.6808 del T, p.Leu2271StopCodon) which was discordant with the clinical symptoms of BMD seen in the patient (dmd28). Meanwhile, one case with a deletion in exon 65 (dmd18) turned out to carry a small in-frame deletion of 12 nucleotides (c. 9540–9551 del CTGGCTGCTGAA, p.Asn3180–3184Asn). This was clinically discordant with the DMD condition observed in the patient ( Table 1). The largest exon deletion in this study spanned exons 3 to 44 (one case). Other large deletions encompassed exons 17–43, exons 7–43, exons 18–47, exons 3–17, exons 48–52, and exons 56–74. The most common type of deletions were deletions of exons 45 to 52, exons 49 to 52, exons 49 to 50, exons 3 to 7 and single deletion of exon 51 (two cases each, respectively) ( Table 1). Deletion mostly occurred in the distal hot spot region (exons 45–55) with 20 cases (66.67%) carrying deletions in this region. In eight cases (26.67%) deletions occurred in the proximal hot spot (exons 2–20) region ( Figures 1 and 2).

### Duplications pattern in the *DMD* gene

**Figure 3.**
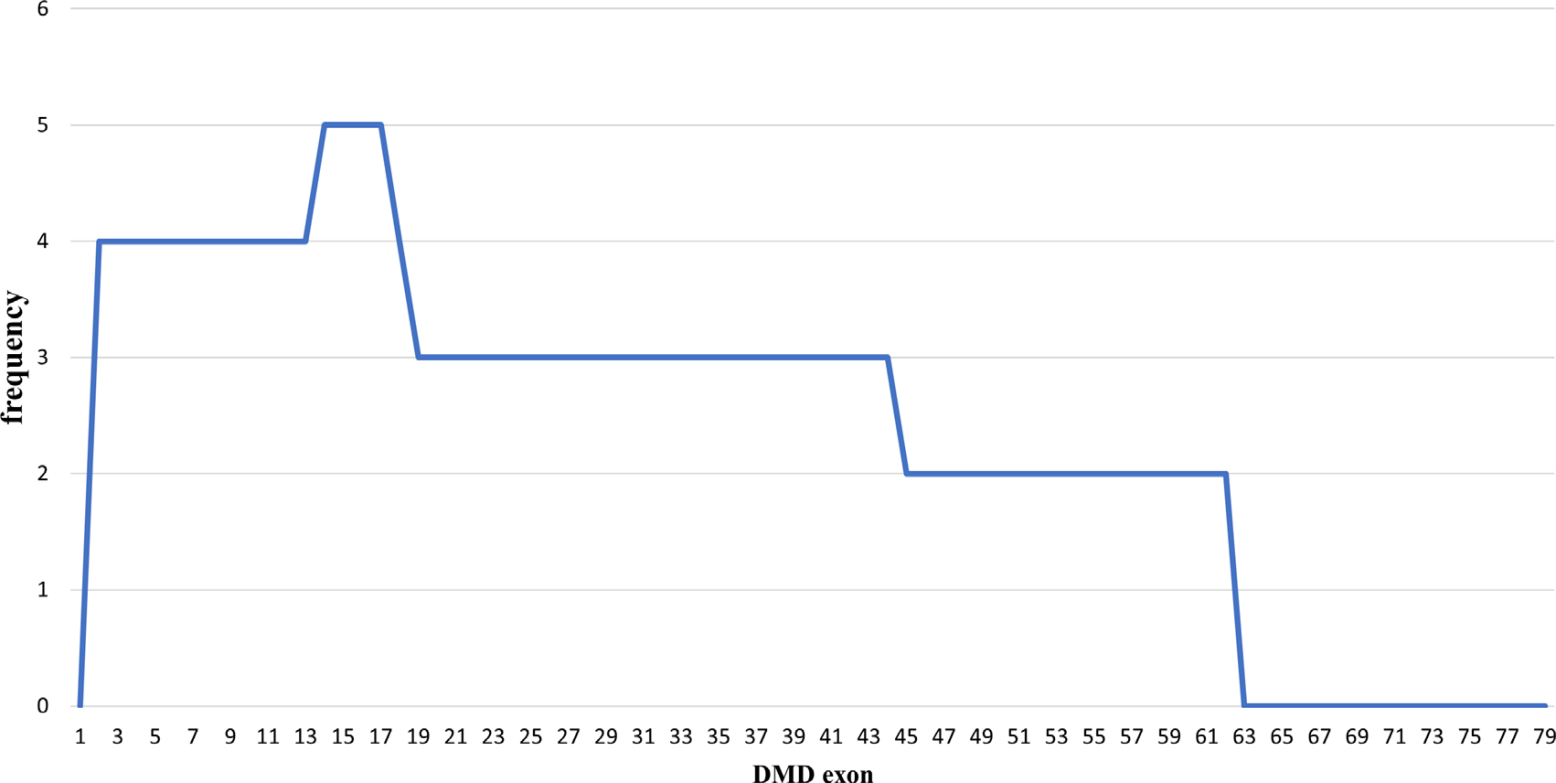
Frequencies of duplication of *DMD* gene exons in Indonesian Duchenne muscular dystrophy/Becker muscular dystrophy patients (n=5).

**Figure 4.**
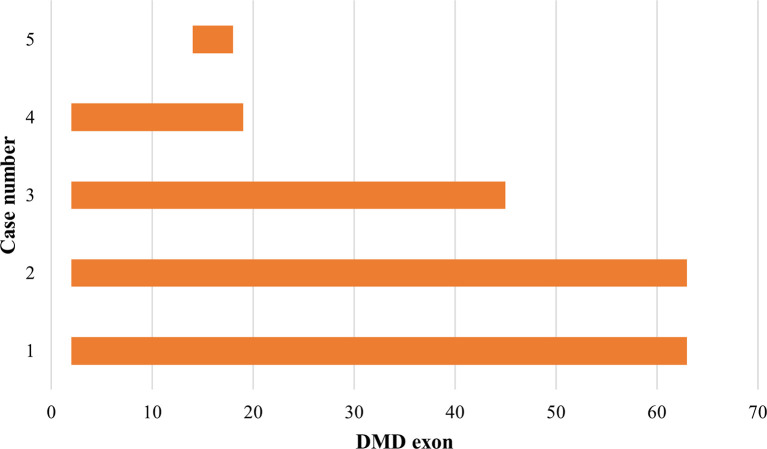
Distribution pattern of exon duplications in Indonesian Duchenne muscular dystrophy/Becker muscular dystrophy patients (n=5). All exon duplications occurred in the rod domain of the *DMD* gene and revealed as out-frame mutations (indicated in orange boxes). In-frame duplication was not detected.

Out of the 43 DMD cases, duplications occurred in five patients (11.63%): four cases had duplications initiating from exon 2, and one case starting from exon 14. The highest frequency of duplications occurred in exons 14 to 17, found in all five patients. The largest duplication spanned from exons 2 to 62 affecting 40% of these cases (two patients), and the shortest duplication covered exons 14 to 17 in 20% (one patient) ( Figures 3 and 4). All duplications (100%) involved the proximal hot spot region.

### Association with reading frames

Out of the 30 patients with deletions, only four cases were in-frame, and the remaining 26 cases were out-of-frame ( Table 1). One discrepancy was dmd31, with a mutation predicted as in-frame (del 3–44); however, the phenotype was DMD. Meanwhile two cases with single exon deletions detected initially by MLPA turned out to carry a nonsense mutation (case dmd28) and a 12-nucleotide deletion (case dmd18) by sequencing. These mutations were out-frame and in-frame respectively. Both cases were discordant with the reading frame predictions as dmd28 showed a milder phenotype whereas dmd18 showed a severe clinical condition ( Table 2). In five patients carrying duplications of the *DMD* gene, all were out-of-frame with DMD phenotype.

Based on immunohistochemical staining results, three cases (dmd20, dmd28 and dmd38) showed negative staining of dystrophin, however the patients manifested milder phenotype of BMD. Meanwhile five cases (dmd10, dmd11, dmd35, dmd39, and dmd54) showed faint staining of dystrophin ( Table 2), even though the genotype–phenotype correlations were consistent with DMD. Among the novel mutations found in this study, two of them (duplication of exon 2 to 62 and deletion of exon 56 to 74) were out-of-frame, while the deletion of exon 7 to 43 was an in-frame mutation ( Table 2). Overall, out of 35 patients with deletion and duplication, 32 patients (91.43%) were in accordance with the reading frame rule, meanwhile three (8.57%) patients were discordant ( Table 2).

### Other clinical parameters

Out of the 43 patients, 18 patients (41.86%) manifested skeletal deformities, including scoliosis in 14 cases (32.56%), lordosis in two cases (4.65%), ankle deformity in two cases (4.65%), coxae deformity in one case (2.32%), knee deformity in one case (2.32%), and lower leg deformity in one case (2.32%). Some patients had more than one skeletal deformity, such as scoliosis coexisting with ankle deformity. One patient (2.32%) was born with low birth weight, three patients (6.98%) suffered from malnutrition and two patients (2.32%) were reported to have cardiomegaly. Six patients (13.85%) showed early signs of respiratory tract disturbance, such as difficulty in breathing and pneumonia. Some patients reported history of milestone developmental problems, including gross motor skill delays in 14 patients (32.56%), fine motor skill delays in seven patients (16.28%) and speech delays in five patients (11.62%) ( Table 1).

### Carrier status

**Figure 5.**
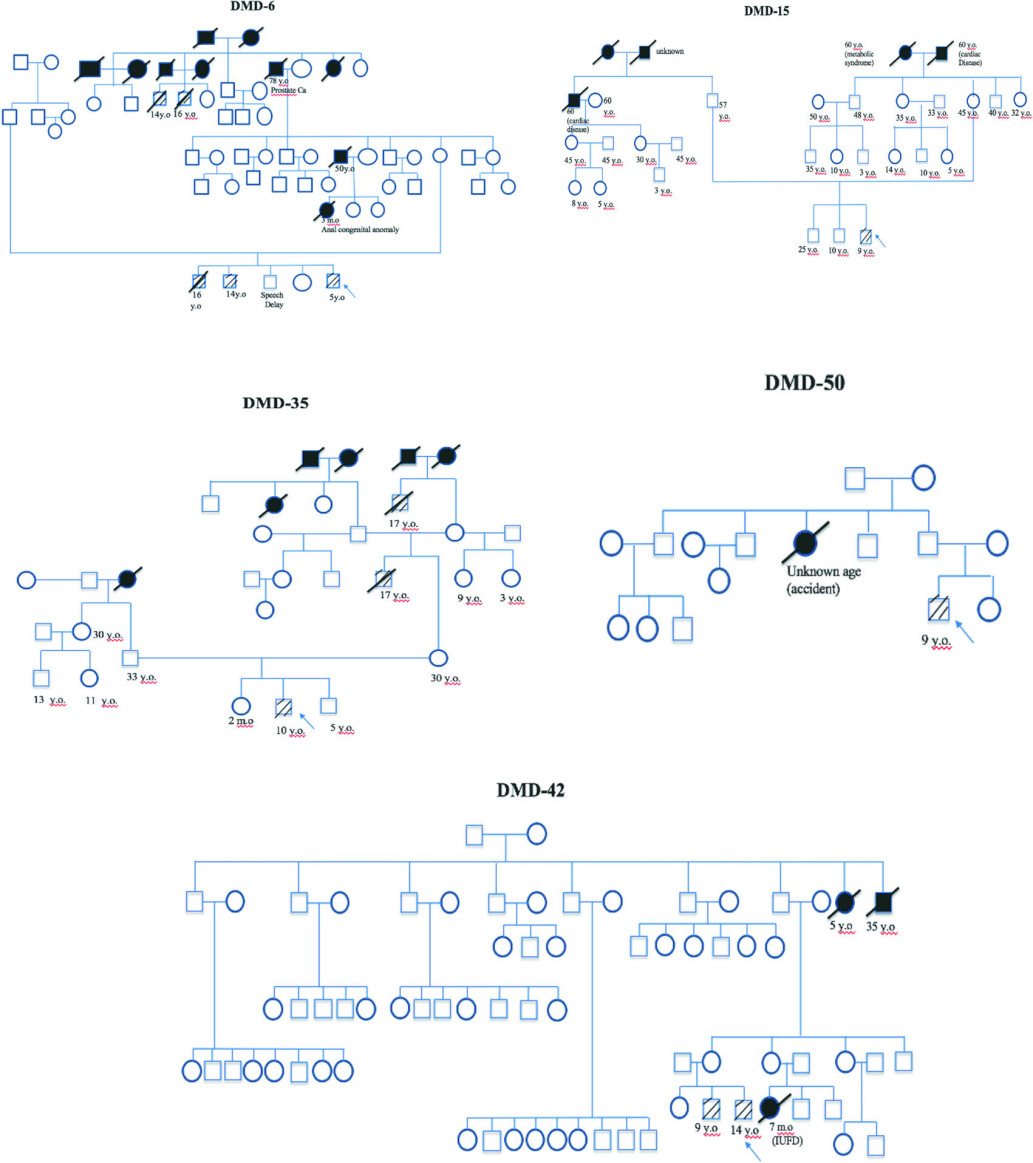
Five family pedigrees of Duchenne muscular dystrophy patients. Arrow indicates index cases, square indicates male, circle indicates female, cross-hatching indicates affected individuals, slashed cross indicates deceased individuals, number indicates age (y.o = years old, m.o = months old), black block indicates unknown cause of death.

Carrier status analysis was performed on mothers and female siblings of patients found to carry deletion or duplication mutations through MLPA analysis. Family members of the patients without deletion or duplication did not undergo this analysis. Twenty-seven available samples from mothers showed that carrier status was confirmed in 14 cases (51.85%). Five available samples from female siblings of probands showed one case (20%) carrying the mutated *DMD* gene, meanwhile the remaining four cases (80%) were negative for the identified mutation in their affected brother. Family pedigrees were available in five patients showing x-linked pattern of inheritance ( Figure 5).

### Molecular therapy applicability

Out of 31 patients whose genotype data was consistent with severe DMD, 60.47% cases are amenable for the application of exon skipping therapy. Therapeutic approaches to skip exons 51, 53, 45, 50, 63, 18, 17, 2, 19, 44, 8 and 55 can be applied in 26 patients (60.47%). One patient with an identified nonsense mutation c.6808 del T can be managed with codon read through therapy. The mutation spectrum and molecular therapy applicability for the identified mutations are presented in Tables 3 and 4.

**Table 3.** Cases amenable for molecular therapies.

Molecular therapies	Type of mutation	Applicable cases	
		n	(%)
**Skipping of**			
Exon 51	del48–50, del52, del47–50, del49–50	5	11.63
Exon 53	del45–52, del48–52, del49–52	5	11.63
Exon 45	del46–48, del46–51	2	4.65
Exon 50	del 51	2	4.65
Exon 63	dup 2–62	2	4.65
Exon 18	dup 14–17, del 3–17	2	4.65
Exon 17	del18–47, del18–34	2	4.65
Exon 2	del3–7	2	4.65
Exon 19	dup2–18	1	2.33
Exon 44	del17–43	1	2.33
Exon 8	del5–7	1	2.33
Exon 55	del56–74	1	2.33
Read through	c.6806delT	1	2.33

**Table 4.** Comparisons of applicability of current molecular therapy reported across different countries (selected examples).

No	Country	Patient number	Del (%)	Dup (%)	Other mutations (%)	No del/No dup (%)	Applicability for current molecular therapies
1	USA (Flanigan *et al.*, 2009)	111	42.9	11	46	(-)	Single exon skipping 59.6%
2	Japan (Takeshima *et al.*, 2011)	442	61	9	29	(-)	single exon skipping 47% (exons 51, 50, 45, 44, 8, 43, 52, 55)
3	India (Polavarapu *et al.*, 2019)	606	81.2	5.4	11.5	1.3	single exon skipping 38.6% (exon 45, 51, 53)
4	Singapore (Tomar *et al.*, 2019)	145	65.5	9.7	21.4	(-)	Single exon skipping 51.6%, multiple exon skipping 32.3%
5	Kuwait (Mohammed *et al.*, 2018)	68	66	5	10	13	Single exon skipping of 16% (exon 51)
6	Eastern European: Poland, Hungary, Lithuania, Romania, Serbia, Croatia, Bosnia, Bulgaria, Ukraine, and Russia (Selvatici *et al.*, 2021)	328	29	11	59	(-)	Single exon skipping 59% (exons 53, 51, 45, 44), 27% for read through
7	Malaysia (Rani *et al.*, 2011)	35	77	6	17	(-)	Single exon skipping 24% (exon 45)
8	Indonesia (this study)	43	65.11	11.63	2.3	23.25	Single exon skipping 60% (exons 51, 53, 45, 50, 17, 2, 44, 8, 55, 63, 18, 19), 2.3% read through

del: deletion; dup: duplication; (-): unknown/data not provided.

## Discussion

In DMD/BMD, the increased permeability of the sarcolemma allows CK from muscle fibers to be released to blood stream, indicating a muscle damage process. The highest levels of CK are commonly observed between two to five years of age and decreases along with disease progression and older age. ^10^ This pattern is similar to what is observed in the DMD/BMD patients in our study, which also showed high CK levels with mean age of onset at five years of age.

The analysis of fresh frozen muscle specimens is usually a standard procedure for muscle biopsy, however this service is not currently available in Indonesia. Instead, FFPE samples from muscle specimens were used to detect dystrophin expression in sarcolemma membrane. Similar use of such FPPE samples have been previously reported in Thailand, Japan and UK, showing this as a reliable and reproducible technique. ^11^ Our immunohistochemistry (IHC) analysis showed weak staining of dystrophin protein in five DMD patients. Trace-level dystrophin expression in patients with out-of-frame DMD mutations has been previously reported in approximately 20% of DMD patients. ^12^^,^^13^ The mechanism for this low level of dystrophin has not been fully understood. Possible mechanisms include re-initiation of translation downstream of frameshift mutation and alternative splicing of exon adjacent to deletion boundaries resulting in restoration of open reading frame. ^14^ Indeed, a study by our group previously showed that the formation of splicing silencer by *DMD* exon 45 deletion junction could explain exon 44 skipping, thereby restoring open reading frame. ^15^ Three other cases with negative dystrophin staining were inconsistent with the BMD genotypes and phenotypes observed. The negative result of dystrophin staining could be due to subjectivity in evaluating the IHC result or insufficient pre-analytical treatment. Moreover, the dystrophin marker used in this study was Dys-2 located in C-terminal, which may not fully capture expression in the rod domain. ^16^ Due to these limitations and the invasive nature of the muscle biopsy procedure, molecular methods of gene analysis from blood draws offer a better option for diagnosis.

The use of the MLPA technique has improved the detection of both single and large intragenic rearrangements because it allows the simultaneous analysis of all 79 exons in the *DMD* gene, the largest gene in the human genome. Prior to the MLPA technique, approaches such as multiplex PCR using primers that cover sets of commonly deleted exons would yield deletion rates ranging from 40% to 51.2% of DMD/BMD cases as reported in some Asian populations. ^17^ *DMD* gene analysis in the Indonesian population has been conducted previously using IHC ^18^ and multiplex PCR ^19^ methods. However, precise mutations cannot be identified using the IHC method while multiplex PCR is unable to cover all exons in the *DMD* gene. Our study using MLPA screening showed that deletions were detected in 69.77% cases while duplications were found in 11.63%, providing a molecular diagnosis in 81.4% of total examined cases. This result is comparable to previous MLPA studies of DMD/BMD cases in Asian populations conducted in China 66–95%, ^20^ India 68%, ^21^ Vietnam (54%), ^22^ Malaysia (77%) ^23^ Japan (70%) ^2^ and Singapore (75%). ^8^ MLPA is suitable to be performed in developing countries as a screening method since it is simple and fast and allows the diagnosis of DMD in about 85% of cases with the occurrence of 60–70% deletions and 10–15% duplications in the *DMD* gene found in all DMD patients. ^1^^,^^21^^,^^24^^–^^26^ This detection will contribute to possible application of exon skipping therapy in 60.47% of DMD cases in our patients, similar to some other studies summarized in Table 4. However, in Kuwait and Malaysia, its applicability is reported to be much lower. ^1^^,^^8^^,^^27^^–^^30^ Therapy using eteplirsen (skipping of exon 51) and golodirsen (skipping of exon 53), antisense oligonucleotide drugs approved by the FDA, can be applied in 23.25% of patients while codon read through therapy using compounds such as ataluren, can be applied in one patient.

A critical issue in interpretation of MLPA results is the detection of deletions involving a single *DMD* exon. In this study, two single exon deletions detected through MLPA turned out to be small point mutations that involve sequences where the probes should ligate. The altered exon sequences inhibited the proper hybridization of the specific probe, thus leading to the observed deletion of the respective exon during MLPA analysis. Such sequence variations may involve pathogenic small or point mutations, or even a polymorphism that does not disrupt gene function. Therefore, single exon deletions detected by MLPA should always be confirmed with PCR and sequencing as evidenced by our observations in this study. ^31^ Two cases (dmd18 and dmd31) in this study were in-frame mutations, but phenotypically DMD. Meanwhile, one case (dmd28) was out-frame in genotype, but phenotypically BMD. Further investigation using mRNA transcripts is needed to reveal the splicing pattern of these cases in order to understand how the discrepancies occurred. Unfortunately, it was not possible to collect additional samples from the patients for further investigations in the current study. Concordance to the reading frame rule in our study was 91.43% (32/35), which is similar to other studies in Asia, including Japan (93%), ^1^ Singapore (96%), ^8^ Vietnam (94%), Malaysia (93%) ^22^ and China (88%). ^20^

In contributing towards the spectrum of mutations in the *DMD* gene, one novel mutation was observed in two patients, dmd19 and dmd21, involving a duplication of exons 2 to 62 (c. (-182_59) (9187_9246) dup). Duplication frequency is reported to be highest at the 5' end of the gene with exon 2 duplication as the most common single duplication. ^1^^,^^32^ This novel mutation in both patients has been deposited into the Leiden Open Variation Database (LOVD) (ID numbers 00383167 and 00383168).

Previous studies on the mutation spectrum of the *DMD* gene in Japan showed that deletions were found to cluster in a proximal hotspot (exon 2–20) and distal hotspot (exon 45–55), at a frequency of 26% and 65% of all identified deletions, respectively. ^1^ This study showed similar results as deletions occurred more frequently in the distal hot spot region (66.67%), and only 26.67% were in the proximal hot spot region. Duplication mutations appear to be distributed across the *DMD* gene; however, the proximal hot spot was more frequently duplicated than the distal hotspot, ^1^ which concurs with our result showing that all duplications (100%) involved the proximal hot spot region.

In this study, the MLPA method was not able to identify deletion nor duplication in eight patients. Small sequence mutations may occur in these cases leading to a negative result in the MLPA since the clinical features were consistent for DMD. Further sequencing analysis using mRNA to detect both point mutation and splicing patterns is needed to reveal the disease-causing mutation. MLPA should be combined with direct Sanger sequencing or whole exome sequencing to obtain higher sensitivity and specificity in the detection of small or point mutations in the *DMD* gene. ^33^ Additionally, it should be noted that MLPA analysis can generate false positive results by showing an exon deletion when a polymorphism occurs at a primer ligation site. ^34^ Hence, it is always useful to conduct a confirmatory test using another method.

Previously, DMD patients commonly lost their ambulation prior to 12 years of age and could only survive until their late teens. ^35^ However, current treatment such as long-term use of corticosteroids has been reported to prolong ambulation by two to five years or even longer, thereby reducing the need of spinal stabilization surgery, improving cardiopulmonary function, delaying the need for noninvasive nasal ventilation, and increasing survival and the quality of life of patients with DMD. ^36^ Surgical and ventilator support were also reported to increase survival up to the third decade. ^37^ In our study, the mean age of losing ambulatory ability was still below 12 years old and this may be caused by the difficulty of the patients obtaining early and proper clinical management due to challenges in establishing precise diagnosis. This is due to difficulty in getting access to molecular testing. Muscle biopsy can be performed to help with diagnosis using immunohistochemistry analysis, ^18^ however, not all medical centers have this facility and not all parents agree to this invasive procedure. Due to such limitations in Indonesia, the disease has not been well characterized. Hence, this current study provides evidence of usefulness of deletion and duplication screening for diagnosis in most patients.

Initially, both DMD and BMD patients show skeletal muscle weakness, marked with positive Gower sign. The natural course of DMD shows that muscular dystrophy will occur progressively followed by deterioration of cardiac and respiratory muscles, leading to early death due to respiratory or heart failure. ^35^ Mutations involving exons 12, 14 to 17, 31 to 42, 45, and 48 to 49 have been reported to enhance cardiac involvement. ^38^ In our study, two patients showed cardiomegaly and only one of them fit with this theory, as this patient had exons 46–50 deletion. Cardiac and respiratory problems were found only in two (2.32%) and six (13.85%) patients, respectively. The small number of patients with cardiac and respiratory problems in this study is perhaps due to the relatively short period of observation. Similar limitations were also encountered in collection of other clinical parameters observed in this study such as musculoskeletal involvement, malnutrition, etc.

Scoliosis is a frequent complication (68–90%) in DMD, meanwhile bone fractures occur in 20–25% of cases and the risk increases along with age and loss of ambulation. ^39^ In our study, cases of skeletal deformity, including scoliosis, were only found in 41.86% of patients, less than previous studies. Some patients were also reported to suffer from malnutrition (6.98%). The weight loss in DMD patients is suspected to be associated with progressive muscle weakness leading to dysphagia and mastication dysfunction. ^40^ Growth delays in individuals with DMD can be observed as short stature ^41^ or motoric and language delays. ^42^ Even though short stature was not observed in all subjects, some patients (34.88%) were found to develop motoric and language delays. Even though dystrophin expression in the brain is only one-tenth of that observed in muscle, varying degrees of non-progressing cognitive impairment might be exhibited in some patients. ^43^ However, no mental retardation was observed in this study.

Theoretically, approximately two-thirds of DMD patients inherit their mutations from carrier mothers, meanwhile the remaining one-third are predicted to develop the disease due to spontaneous mutations. ^44^ Our results showed that inherited cases were confirmed in 14 cases (51.85%) out of 27 available samples while 13 cases were due to *de novo* mutations (48.15%). These results suggest that the rate of *de novo* mutation in our population is higher than the estimated one-third of all DMD cases. However, there has been one previous study of 150 cases showing a high rate (71%) of *de novo* mutations as compared to the inherited cases which affected only 49 cases (29%). ^45^ *De novo* mutations are defined by their presence in one or more progenies and the absence in the mother. The presence of multiple affected off-spring from apparently non-carrier parents is caused by germline mosaicism as *DMD* mutation evolves *de novo* in the affected patient and the risk of recurrence from germinal mosaicism is estimated to be approximately 4.3%. ^46^

MLPA has been used to detect carrier status in probands with known deletion or duplication mutations since it is an easy and quick technique. ^47^ Detection of carrier status serves to provide essential information for family counselling regarding future pregnancies, improving quality of life and reducing financial burden for at-risk families. ^48^ This study also shows that MLPA analysis can pave the way for future therapeutics in DMD patients by identifying amenable genotypes that can be targeted.

Mutation detection in DMD is essential for patient management especially since advanced therapy such as exon skipping, CRISPR-Cas9 mediated correction or stop codon read-through therapy are mutation specific approaches. Furthermore, molecular diagnosis is also important for genetic counselling to plan future pregnancy. In our study, MLPA was found to be an easy and quick technique to identify deletion and/or duplication mutation and detect copy number of DMD carriers. This study also showed that the majority (81.40%) of *DMD* gene mutations in Indonesian DMD patients were deletions and duplications. Of these, 60.46% would be amenable to exon skipping therapy.

This is the first study showing the feasibility of implementing the MLPA method in detecting DMD gene mutation and revealing the spectrum of common mutations in Indonesia. This is also the first study showing the potential application of exon skipping therapy in the majority of DMD cases in the country. The clinical and molecular characterization of patients in this study provide a better insight of DMD and BMD profiles in the Indonesian population and will shape health policies in patient management.

## Data availability

### Underlying data

Figshare: Underlying data for ‘Mutation spectrum analysis of *DMD* gene in Indonesian Duchenne and Becker muscular dystrophy patients’, https://www.doi.org/10.6084/m9.figshare.15172167. ^9^

This project contains the following underlying data:

• Demographic, clinical and genetic characteristic of Duchenne muscular dystrophy (DMD)/Becker muscular dystrophy (BMD) patients
• Electrophoresis result of single exon deletion of exon 47, exon 51, and exon 52.
• Electrophoresis result of single exon deletion of exon 47 and exon 65.

Data are available under the terms of the Creative Commons Zero “No rights reserved” data waiver (CC0 1.0 Public domain dedication).

## Accession numbers ^*^

Leiden Open Variation Database (LOVD): DMD variant (del 46–48). Accession number DMD_014648, https://databases.lovd.nl/shared/view/DMD?search_VariantOnGenome%2FDBID=%3D%22DMD_014648%22

Leiden Open Variation Database (LOVD): DMD variant (del 53–54). Accession number DMD_105354, https://databases.lovd.nl/shared/view/DMD?search_VariantOnGenome%2FDBID=%3D%22DMD_015354%22

Leiden Open Variation Database (LOVD): DMD variant (del 46–50). Accession number DMD_014650, https://databases.lovd.nl/shared/view/DMD?search_VariantOnGenome%2FDBID=%3D%22DMD_014650%22

Leiden Open Variation Database (LOVD): DMD variant (del 17–43). Accession number DMD_011743, https://databases.lovd.nl/shared/view/DMD?search_VariantOnGenome%2FDBID=%3D%22DMD_011743%22

Leiden Open Variation Database (LOVD): DMD variant (del 45–52). Accession number DMD_014552, https://databases.lovd.nl/shared/view/DMD?search_VariantOnGenome%2FDBID=%3D%22DMD_014552%22

Leiden Open Variation Database (LOVD): DMD variant (del 51). Accession number DMD_015151, https://databases.lovd.nl/shared/view/DMD?search_VariantOnGenome%2FDBID=%3D%22DMD_015151%22

Leiden Open Variation Database (LOVD): DMD variant (del 46–51). Accession number DMD_014651, https://databases.lovd.nl/shared/view/DMD?search_VariantOnGenome%2FDBID=%3D%22DMD_014651%22

Leiden Open Variation Database (LOVD): DMD variant (del 48–50). Accession number DMD_014850, https://databases.lovd.nl/shared/view/DMD?search_VariantOnGenome%2FDBID=%3D%22DMD_014850%22

Leiden Open Variation Database (LOVD): DMD variant (del 52). Accession number DMD_015252, https://databases.lovd.nl/shared/view/DMD?search_VariantOnGenome%2FDBID=%3D%22DMD_015252%22

Leiden Open Variation Database (LOVD): DMD variant (del 65). Accession number DMD_016565, https://databases.lovd.nl/shared/view/DMD?search_VariantOnGenome%2FDBID=%3D%22DMD_016565%22

Leiden Open Variation Database (LOVD): DMD variant (dup 2–62). Accession number DMD_020262, https://databases.lovd.nl/shared/view/DMD?search_VariantOnGenome%2FDBID=%3D%22DMD_020262%22

Leiden Open Variation Database (LOVD): DMD variant (del 7–43). Accession number DMD_010743, https://databases.lovd.nl/shared/view/DMD?search_VariantOnGenome%2FDBID=%3D%22DMD_010743%22

Leiden Open Variation Database (LOVD): DMD variant (del 47–50). Accession number DMD_014750, https://databases.lovd.nl/shared/view/DMD?search_VariantOnGenome%2FDBID=%3D%22DMD_014750%22

Leiden Open Variation Database (LOVD): DMD variant (del 49–52). Accession number DMD_014952, https://databases.lovd.nl/shared/view/DMD?search_VariantOnGenome%2FDBID=%3D%22DMD_014952%22

Leiden Open Variation Database (LOVD): DMD variant (del 47). Accession number DMD_014747, https://databases.lovd.nl/shared/view/DMD?search_VariantOnGenome%2FDBID=%3D%22DMD_014747%22

Leiden Open Variation Database (LOVD): DMD variant (del 51–54). Accession number DMD_015154, https://databases.lovd.nl/shared/view/DMD?search_VariantOnGenome%2FDBID=%3D%22DMD_015154%22

Leiden Open Variation Database (LOVD): DMD variant (del 3–44). Accession number DMD_010344, https://databases.lovd.nl/shared/view/DMD?search_VariantOnGenome%2FDBID=%3D%22DMD_010344%22

Leiden Open Variation Database (LOVD): DMD variant (del 49–50). Accession number DMD_014950, https://databases.lovd.nl/shared/view/DMD?search_VariantOnGenome%2FDBID=%3D%22DMD_014950%22

Leiden Open Variation Database (LOVD): DMD variant (dup 14–17). Accession number DMD_021417, https://databases.lovd.nl/shared/view/DMD?search_VariantOnGenome%2FDBID=%3D%22DMD_021417%22

Leiden Open Variation Database (LOVD): DMD variant (del 18–47). Accession number DMD_011847, https://databases.lovd.nl/shared/view/DMD?search_VariantOnGenome%2FDBID=%3D%22DMD_011847%22

Leiden Open Variation Database (LOVD): DMD variant (del 56–74). Accession number DMD_015674, https://databases.lovd.nl/shared/view/DMD?search_VariantOnGenome%2FDBID=%3D%22DMD_011847%22

Leiden Open Variation Database (LOVD): DMD variant (del 45–49). Accession number DMD_014549, https://databases.lovd.nl/shared/view/DMD?search_VariantOnGenome%2FDBID=%3D%22DMD_014549%22

Leiden Open Variation Database (LOVD): DMD variant (del 18–34). Accession number DMD_011834, https://databases.lovd.nl/shared/view/DMD?search_VariantOnGenome%2FDBID=%3D%22DMD_011834%22

Leiden Open Variation Database (LOVD): DMD variant (dup 2–44). Accession number DMD_020244, https://databases.lovd.nl/shared/view/DMD?search_VariantOnGenome%2FDBID=%3D%22DMD_020244%22

Leiden Open Variation Database (LOVD): DMD variant (del 48–52). Accession number DMD_014852, https://databases.lovd.nl/shared/view/DMD?search_VariantOnGenome%2FDBID=%3D%22DMD_014852%22

Leiden Open Variation Database (LOVD): DMD variant (del 3–7). Accession number DMD_010307, https://databases.lovd.nl/shared/view/DMD?search_VariantOnGenome%2FDBID=%3D%22DMD_010307%22

Leiden Open Variation Database (LOVD): DMD variant (del 5–7). Accession number DMD_010507, https://databases.lovd.nl/shared/view/DMD?search_VariantOnGenome%2FDBID=%3D%22DMD_010507%22

Leiden Open Variation Database (LOVD): DMD variant (del 3–17). Accession number DMD_010317, https://databases.lovd.nl/shared/view/DMD?search_VariantOnGenome%2FDBID=%3D%22DMD_010317%22

Leiden Open Variation Database (LOVD): DMD variant (dup 2–18). Accession number DMD_020218, http://www.umd.be/DMD/4DACTION/WV/368

## Consent

Written informed consent for publication of the patients’ details was obtained from the parents of the patients.

## Footnotes

^*^
Representative Accession numbers are shown as some variants have been associated with numerous IDs.
