# Supplementary material for: Mutation spectrum analysis of DMD gene in Indonesian Duchenne and Becker muscular dystrophy patients
Source: F1000Res. 2023 Nov 17;11:148. Originally published 2022 Feb 7. [Version 3] doi: 10.12688/f1000research.73476.3 (PMC10668572; doi:10.12688/f1000research.73476.3)
Supplement: Peer review history

# Reviewer report: Reviewer response for version 3

**DOI:** 10.5256/f1000research.158228.r223671

**Published:** 2023-11-22

**Authors:** Luca Bello

**Affiliations:** 1 University of Padova, Padova, Italy

The authors have responded satisfactorily to my comments.

Is the work clearly and accurately presented and does it cite the current literature?

Yes

If applicable, is the statistical analysis and its interpretation appropriate?

Yes

Are all the source data underlying the results available to ensure full reproducibility?

Yes

Is the study design appropriate and is the work technically sound?

Yes

Are the conclusions drawn adequately supported by the results?

Yes

Are sufficient details of methods and analysis provided to allow replication by others?

Yes

Reviewer Expertise:

Muscular dystrophy

I confirm that I have read this submission and believe that I have an appropriate level of expertise to confirm that it is of an acceptable scientific standard.

# Reviewer report: Reviewer response for version 2

**DOI:** 10.5256/f1000research.143201.r184879

**Published:** 2023-09-06

**Authors:** Luca Bello

**Affiliations:** 1 University of Padova, Padova, Italy

This is a rigorously conducted and well-written study describing the “genetic architecture” of dystrophinopathy in 43 patients from two neuromuscular Centers in an Indonesian city. The literature is quite rich of such papers, showing mutational composition of dystrophinopathy cohorts from different world populations, and these are indeed, as the authors claim, useful for directing local health interventions and policies. However, it would be in my opinion also useful to point out that, in sufficiently large populations, the overall genetic composition of dystrophinopathies tends to be very similar across world populations, as there are no or very few “founder” mutations (because of the severe phenotype and X-linked nature) and most, if not all identified mutations, usually date back to just a few generations.

 Moreover, I have some comments and suggestions, as listed below:

1. Table 3: Out-of-frame duplications are not amenable to exon skipping.
2. Also skipping of exon 55 for del 56-74 is very theoretical (no dystroglycan binding in the expected protein product).
3. c.6808delT is not a nonsense mutation, but rather a frameshift deletion of 1 nucleotide; therefore a DMD phenotype is expected. The fact that the patient walks at age 9 (with corticosteroid treatment?) does not exclude a DMD phenotype. This patient should **not **be treated with ataluren.
4. How many of the 43 cases are related cases (siblings, cousins, other)? This point is also relevant for the discussion of de novo frequency in the population (the more siblings, the less de novo).
5. The 12-nucleotide deletion in exon 65 may disrupt binding to β-dystroglycan, a potential explanation for a severe phenotype despite ORF preservation in this patient.
6. Figure 5 (pedigrees) is not very useful, as X-linked inheritance is expected and always confirmed.
7. “Gowers” instead of “Gower”.
8. Are the Centers where the data were collected wxclusively pediatric? This may exclude from this population the spectrum of milder BMD which only becomes clinically evident in adult age, and should be considered in the discussion (i.e. underestimation of in-frame mutations).
9. For reference 3 I would suggest Monaco *et al.* (1988 ^1^), rather than, or in addition to, the current reference.

Is the work clearly and accurately presented and does it cite the current literature?

Yes

If applicable, is the statistical analysis and its interpretation appropriate?

Yes

Are all the source data underlying the results available to ensure full reproducibility?

Yes

Is the study design appropriate and is the work technically sound?

Yes

Are the conclusions drawn adequately supported by the results?

Yes

Are sufficient details of methods and analysis provided to allow replication by others?

Yes

Reviewer Expertise:

Muscular dystrophy

I confirm that I have read this submission and believe that I have an appropriate level of expertise to confirm that it is of an acceptable scientific standard, however I have significant reservations, as outlined above.

## References

[1] : An explanation for the phenotypic differences between patients bearing partial deletions of the DMD locus. *Genomics* .1988;2(1) : 10.1016/0888-7543(88)90113-9 90-95 10.1016/0888-7543(88)90113-9 3384440

# Response

**Published:** 2023-11-03

**Authors:** Poh San Lai

**Affiliations:** National University of Singapore, Singapore

**Response to reviewer:**

*1. Table 3: Out-of-frame duplications are not amenable to exon skipping.*

 Response: Thank you for the comment. We have amended Table 3 to remove this.

*2. Also skipping of exon 55 for del 56-74 is very theoretical (no dystroglycan binding in the expected protein product).*

 Response: Thank you for the comment. We have amended Table 3 to remove this.  

*3. c.6808delT is not a nonsense mutation, but rather a frameshift deletion of 1 nucleotide; therefore a DMD phenotype is expected. The fact that the patient walks at age 9 (with corticosteroid treatment?) does not exclude a DMD phenotype. This patient should **not **be treated with ataluren.*

 Response: Thank you for pointing out the error. We agree that ataluren will not affect the patient with the frameshift deletion and have revised Table 3.  

*4. How many of the 43 cases are related cases (siblings, cousins, other)? This point is also relevant for the discussion of de novo frequency in the population (the more siblings, the less de novo).*

 Response: Thank you for the comment. We checked again and none of related cases was included in this study.

*5. The 12-nucleotide deletion in exon 65 may disrupt binding to β-dystroglycan, a potential explanation for a severe phenotype despite ORF preservation in this patient.*

 Response: Thank you very much for your insight. We have added this to the 4 ^th^ paragraph of the discussion section. All changes are highlighted in yellow in the manuscript text.

*6. Figure 5 (pedigrees) is not very useful, as X-linked inheritance is expected and always confirmed.*

 Response: Thank you for the suggestion, we have removed Figure 5 from the revised manuscript.

*7. “Gowers” instead of “Gower”.*

 Response: Thank you for pointing this out. We have made the revision.

*8. Are the Centers where the data were collected exclusively pediatric? This may exclude from this population the spectrum of milder BMD which only becomes clinically evident in adult age and should be considered in the discussion (i.e. underestimation of in-frame mutations).*

 Response: Thank you very much for this important point. We agree and have added this point in the penultimate paragraph of the discussion section.

*9. For reference 3 I would suggest Monaco et al. (1988* *^1^* *), rather than, or in addition to, the current reference.*

 Response: Thank you for the suggestion. We have revised reference 3.

 10. *“* *However, it would be in my opinion also useful to point out that, in sufficiently large populations, the overall genetic composition of dystrophinopathies tends to be very similar across world populations, as there are no or very few “founder” mutations (because of the severe phenotype and X-linked nature) and most, if not all identified mutations, usually date back to just a few generations”.*

 Response: We have added this suggested comment in the discussion section as “Nonetheless, it should be noted that when sufficiently large populations of patients are analyzed, the overall genetic composition of dystrophinopathies tends to be very similar across world populations, as there are no or very few “founder” mutations (because of the severe phenotype and X-linked nature) and most, if not all identified mutations, usually date back to just a few generations”.

# Reviewer report: Reviewer response for version 2

**DOI:** 10.5256/f1000research.143201.r162602

**Published:** 2023-04-25

**Authors:** Teguh Haryo Sasongko

**Affiliations:** 1 Center for Research Excellence, Perdana University-RCSI, Serdang, Malaysia; 2 Graduate School of Medicine, Perdana University-RCSI, Serdang, Malaysia

The authors have sufficiently addressed all concerns. No further comment.

Is the work clearly and accurately presented and does it cite the current literature?

Yes

If applicable, is the statistical analysis and its interpretation appropriate?

Not applicable

Are all the source data underlying the results available to ensure full reproducibility?

No

Is the study design appropriate and is the work technically sound?

Yes

Are the conclusions drawn adequately supported by the results?

Yes

Are sufficient details of methods and analysis provided to allow replication by others?

No

Reviewer Expertise:

Human Molecular Genetics and Rare Diseases

I confirm that I have read this submission and believe that I have an appropriate level of expertise to confirm that it is of an acceptable scientific standard.

# Reviewer report: Reviewer response for version 1

**DOI:** 10.5256/f1000research.77130.r122827

**Published:** 2022-03-22

**Authors:** Teguh Haryo Sasongko

**Affiliations:** 1 Center for Research Excellence, Perdana University-RCSI, Serdang, Malaysia; 2 Graduate School of Medicine, Perdana University-RCSI, Serdang, Malaysia

The authors described their findings on the mutations of DMD gene in Indonesian patients with Duchenne/Becker Muscular Dystrophy. This the first report from Indonesia on the use of MLPA for the molecular diagnosis of DMD, well-written, and with balance clinical, pathological and molecular data.

 There are a few concerns raised:

1. I believe clinical diagnosis was the basis for patients' inclusion into the study. There are 8 patients without evident deletion or duplication. There should be enough information how these patients were clinically categorized into DMD or BMD or they were simply MD pending molecular confirmation (eg. (a) if the clinical diagnosis/examination was made by qualified neurologist/pediatrician (b) information on dmd22 and dmd26 may be very limited to warrant specific clinical diagnosis of DMD. There was no evident loss of dystrophin in these patients).
2. Page 4 mentioned that databases were accessed on 6 June 2021. It will be worthwhile to have an updated comparison as almost a year has lapsed.
3. Table 1 on the column "Age when wheelchair bound", it will be clearer to replace "not yet" with "still ambulant".
4. Table 2 is redundant as the content would have been explained by Table 1.
5. Table 3 and 4 may be combined to focus on discrepancy findings (genotype vs phenotype; IHC vs phenotype).

Is the work clearly and accurately presented and does it cite the current literature?

Yes

If applicable, is the statistical analysis and its interpretation appropriate?

Not applicable

Are all the source data underlying the results available to ensure full reproducibility?

No

Is the study design appropriate and is the work technically sound?

Yes

Are the conclusions drawn adequately supported by the results?

Yes

Are sufficient details of methods and analysis provided to allow replication by others?

No

Reviewer Expertise:

Human Molecular Genetics and Rare Diseases

I confirm that I have read this submission and believe that I have an appropriate level of expertise to confirm that it is of an acceptable scientific standard, however I have significant reservations, as outlined above.

# Response

**Published:** 2023-01-13

**Authors:** Poh San Lai

**Affiliations:** National University of Singapore, Singapore

1. I believe clinical diagnosis was the basis for patients' inclusion into the study. There are 8 patients without evident deletion or duplication. There should be enough information how these patients were clinically categorized into DMD or BMD or they were simply MD pending molecular confirmation (eg. (a) if the clinical diagnosis/examination was made by qualified neurologist/pediatrician (b) information on dmd22 and dmd26 may be very limited to warrant specific clinical diagnosis of DMD. There was no evident loss of dystrophin in these patients).

Response :

 a. The diagnosis was made by qualified pediatric neurologists in our hospital, with enough years of experiences and also hold degree of PhD in area of genetic and muscular dystrophy. We have added explanation about this in the method section.

 b. DMD/BMD are the most common muscular dystrophy with the patients showing gower signs, difficulty in walking, increase CK level, AST or ALT at clinical level. This clinical screening was recommended by previous study as well (1, 2). Male-young patients with those clinical condition may have high possibility of having *DMD* gene mutation and enough reason to do screening with either immunohistochemistry (from tissue biopsy) or MLPA (form blood).

 Unfortunately for patient DMD22 and DMD26, the muscle biopsy was not performed due to consent problem as it is an invasive procedure. Even though immunohistochemistry result was not available, and genetic analysis of MLPA did not reveal any mutation, we believe the clinical data is enough to include the patients. Further tests need to be done to identify  possible small mutations using DNA sequencing. We explained about this in the 7 ^th^ paragraph of discussion section (highlighted).

 2. Page 4 mentioned that databases were accessed on 6 June 2021. It will be worthwhile to have an updated comparison as almost a year has lapsed.

 Response: This is an important suggestion. However due to technical difficulties, especially during the pandemic situation, we have been unable to perform a comprehensive database update. Therefore our most current data is from 6 June 2021. 

 3. Table 1 on the column "Age when wheelchair bound", it will be clearer to replace "not yet" with "still ambulant".

 Response : Thank you very much for this suggestion. The suggestion has been incorporated in our manuscript.

 4. Table 2 is redundant as the content would have been explained by Table 1.

 Response : Thank you very much for this suggestion, this table has been removed. Table numbers have been updated accordingly throughout the manuscript.

 Table 3 and 4 may be combined to focus on discrepancy findings (genotype vs phenotype; IHC vs phenotype).

 Response: Thank you very much for this suggestion, table 3 and 4 have been combined. Table numbers have been updated accordingly throughout the manuscript.

# Response

**Published:** 2023-10-28

**Authors:** Poh San Lai

**Affiliations:** National University of Singapore, Singapore

Response to reviewer

 1.    Table 3: Out-of-frame duplications are not amenable to exon skipping.

 Response:  Thank you for the comment. We have amended Table 3 to remove this.

 2.    Also skipping of exon 55 for del 56-74 is very theoretical (no dystroglycan binding in the expected protein product).

 Response: Thank you for the comment. We have amended Table 3 to remove this.  

 3.    c.6808delT is not a nonsense mutation, but rather a frameshift deletion of 1 nucleotide; therefore a DMD phenotype is expected. The fact that the patient walks at age 9 (with corticosteroid treatment?) does not exclude a DMD phenotype. This patient should not be treated with ataluren.

 Response: Thank you for pointing out the error. We agree that ataluren will not affect the patient with the frameshift deletion and have revised Table 3.  

 4.    How many of the 43 cases are related cases (siblings, cousins, other)? This point is also relevant for the discussion of de novo frequency in the population (the more siblings, the less de novo).

 Response: Thank you for the comment. We checked again and none of related cases was included in this study.

 5.    The 12-nucleotide deletion in exon 65 may disrupt binding to β-dystroglycan, a potential explanation for a severe phenotype despite ORF preservation in this patient.

 Response: Thank you very much for your insight. We have added this to the 4th paragraph of the discussion section. All changes are highlighted in yellow in the manuscript text. 

 6.    Figure 5 (pedigrees) is not very useful, as X-linked inheritance is expected and always confirmed.

 Response: Thank you for the suggestion, we have removed Figure 5 from the revised manuscript. 

 7.    “Gowers” instead of “Gower”.

 Response: Thank you for pointing this out. We have made the revision. 

 8.    Are the Centers where the data were collected exclusively pediatric? This may exclude from this population the spectrum of milder BMD which only becomes clinically evident in adult age and should be considered in the discussion (i.e. underestimation of in-frame mutations).

 Response: Thank you very much for this important point. We agree and have added this point in the penultimate paragraph of the discussion section. 

 9.    For reference 3 I would suggest Monaco et al. (19881), rather than, or in addition to, the current reference.

 Response: Thank you for the suggestion. We have revised reference 3.

 10. “However, it would be in my opinion also useful to point out that, in sufficiently large populations, the overall genetic composition of dystrophinopathies tends to be very similar across world populations, as there are no or very few “founder” mutations (because of the severe phenotype and X-linked nature) and most, if not all identified mutations, usually date back to just a few generations”. 

 Response: We have added this suggested comment in the discussion section as “Nonetheless, it should be noted that when sufficiently large populations of patients are analyzed, the overall genetic composition of dystrophinopathies tends to be very similar across world populations, as there are no or very few “founder” mutations (because of the severe phenotype and X-linked nature) and most, if not all identified mutations, usually date back to just a few generations”.
